# The Potential Protective Role of Caveolin-1 in Intestinal Inflammation in TNBS-Induced Murine Colitis

**DOI:** 10.1371/journal.pone.0119004

**Published:** 2015-03-10

**Authors:** Carolyn R. Weiss, Qingdong Guan, Yanbing Ma, Gefei Qing, Charles N. Bernstein, Richard J. Warrington, Zhikang Peng

**Affiliations:** 1 Department of Immunology, University of Manitoba, Winnipeg, Manitoba, Canada; 2 Department of Pediatrics and Child Health, University of Manitoba, Winnipeg, Manitoba, Canada; 3 Department of Pathology, University of Manitoba, Winnipeg, Manitoba, Canada; 4 Department of Internal Medicine, University of Manitoba, Winnipeg, Manitoba, Canada; 5 IBD Clinical and Research Centre, University of Manitoba, Winnipeg, Manitoba, Canada; Charité, Campus Benjamin Franklin, GERMANY

## Abstract

**Background:**

Caveolin-1 (Cav-1) is a multifunctional scaffolding protein serving as a platform for the cell’s signal-transduction and playing an important role in inflammation. However, its role in inflammatory bowel disease is not clear. A recent study showed that Cav-1 is increased and mediates angiogenesis in dextran sodium sulphate-induced colitis, which are contradictory to our pilot findings in 2,4,6-trinitrobenzene sulphonic acid (TNBS)-induced colitis. In the present study, we further clarified the role of Cav-1 in TNBS-induced colitis.

**Methods:**

In BALB/c mice, acute colitis was induced by intra-rectal administration of one dose TNBS, while chronic colitis was induced by administration of TNBS once a week for 7 weeks. To assess the effects of complete loss of Cav-1, Cav-1 knockout (Cav-1^−/−^) and control wild-type C57 mice received one TNBS administration. Body weight and clinical scores were monitored. Colon Cav-1 and pro-inflammatory cytokine levels were quantified through ELISAs. Inflammation was evaluated through histological analysis.

**Results:**

Colon Cav-1 levels were significantly decreased in TNBS-induced colitis mice when compared to normal mice and also inversely correlated with colon inflammation scores and proinflammatory cytokine levels (IL-17, IFN-γ and TNF) significantly. Furthermore, after administration of TNBS, Cav-1^−/−^ mice showed significantly increased clinical and colon inflammatory scores and body weight loss when compared with control mice.

**Conclusions and Significance:**

Cav-1 may play a protective role in the development of TNBS-induced colitis. Our findings raise an important issue in the evaluation of specific molecules in animal models that different models may exhibit opposite results because of the different mechanisms involved.

## Introduction

Inflammatory bowel disease (IBD), which includes Crohn’s disease and ulcerative colitis, is a chronic remitting and relapsing inflammatory condition of the gastrointestinal tract. The incidence and prevalence of IBD have markedly increased in recent years. Estimates indicate that IBD affects about 1.0–1.5 million Americans [1]. Population-based data from a five province study in Canada indicate that approximately 0.5% of Canadians have IBD (translating to about 170,000 individuals), and incidence rates across the country are among the highest in the world [2]. Although the cause of IBD remains unknown, studies have provided evidence that the pathogenesis of IBD is associated with genetic and environmental factors, enteric flora, and immunological abnormalities [3, 4]. A dysregulation of mucosal immunity in the gut causes an overproduction of pro-inflammatory cytokines and aggregation of immune cells in intestinal mucosa thus leading to uncontrolled mucosal inflammation [5]. Crohn’s disease is caused by an overly aggressive T helper type 1 (Th1) immune response and, as recently found, an excessive IL-23/Th17 pathway activation by bacterial antigens in genetically predisposed individuals [3, 5–7]. Ulcerative colitis, on the other hand, is more of a Th2-like disease with overproduced IL-5 and IL-13 and decreased IFN-γ [8]. As the cause of IBD still remains unknown, the search for new molecules involved with the pathogenesis is ongoing.

Caveolae were first discovered in the 1950s and observed as small, 50–100 nm, cave-like invaginations in the plasma membrane. Prominent in many different types of cells (such as endothelial cells and fibroblasts), these specialized lipid rafts act as cell signalling platforms and regulate the kinetics of vesicle transport by concentrating or segregating receptors and signalling intermediates to form a microenvironment [9–11]. Caveolins require certain structural components for formation: caveolin-1 (Cav-1), caveolin-2, and caveolin-3. Each of these coat proteins have specific roles which can vary from cell type to cell type [12].

Cav-1 has been associated with a number of biological roles in various disease conditions. Using a well-established sepsis animal model, Cav-1 knockout mice showed prolonged and uncontrolled cytokine generation and increased bacterial burden, suggesting that Cav-1 may be a critical protective modulator in animal sepsis [13]. Other studies have shown that Cav-1 may have a tumour suppressive role. Cav-1 has been shown to inhibit a number of oncogenic signalling pathways and function as a tumour/transformation suppressor [14]. In the lung, Cav-1 markedly ameliorated pulmonary fibrosis [15], airway remodeling [16], and was beneficial in the fibrotic phase of lung injury [17]. Thus, Cav-1 has long been thought to play a protective role in the inflammatory response.

However, other studies indicate that Cav-1 may be a potential therapeutic target [18]. A high level of Cav-1 expression is associated with metastatic progression of human prostate cancer [19] and other cancers [20]. Evidence also suggests that Cav-1 may be involved in diabetes-associated inflammation [21, 22], atherosclerosis [23], and cardiovascular diseases [24, 25]. Until now, only one study reported the role of Cav-1 in experimental colitis [26]. There it was observed that Cav-1 was up-regulated during dextran sodium sulphate (DSS)-induced murine colitis and a loss of Cav-1 significantly protected against inflammatory tissue damage. So, it was concluded endothelial Cav-1 mediates angiogenesis in experimental colitis, suggesting that Cav-1 might be a novel therapeutic target for IBD [26].

Our group has developed vaccines against IL-12, IL-23 and TGB-β for the treatment of IBD and evaluated the effects of these vaccines in 2,4,6-trinitrobenzene sulphonic acid (TNBS)-induced experimental mouse colitis [27–29], a colitis similar to human Crohn’s disease. Thus, our original plan was to develop a vaccine targeting Cav-1 and, hopefully, alleviating colitis symptoms. To our surprise, Cav-1 levels were significantly reduced in mice with TNBS-induced colitis. In the present study, we reported this finding and analyzed the relationship of Cav-1 and colonic inflammation. Furthermore, we investigated whether total loss of Cav-1 would worsen colonic inflammation in Cav-1 knockout mice, to confirm our finding. Thus, we concluded that Cav-1 may play an important role in protection from TNBS-induced colitis. As both reports of TNBS- and DSS-induced colitis studied Cav-1 knockout mice, providing evidence of opposite effects, it is critical to be aware that in the evaluation of specific molecules in animal models, there may be opposite results dependent on the pathogenetic mechanisms that are involved.

## Materials and Methods

### Animals

Female Balb/c mice (7–8 weeks old) and female and male C57BL/6J mice (10–12 weeks) were purchased from Charles River Laboratories (Saint-Constant, Quebec, Canada). Female C57BL/6JBL/6J Cav-1 knock-out mice (Cav1^tm1Mls^/J, 7–8 weeks old) and control C57BL/6J (Wild-type B6129SF2/J, 7–8 weeks) mice were purchased from Jackson Laboratories (Bar Harbor, Maine). They were maintained at the Central Animal Care Services, University of Manitoba. All protocols used were approved by the Bannatyne Animal Care Committee, Animal Care & Veterinary Services, University of Manitoba (Protocol #13-055).

### Protocols for induction of colitis

**Fig 1:**
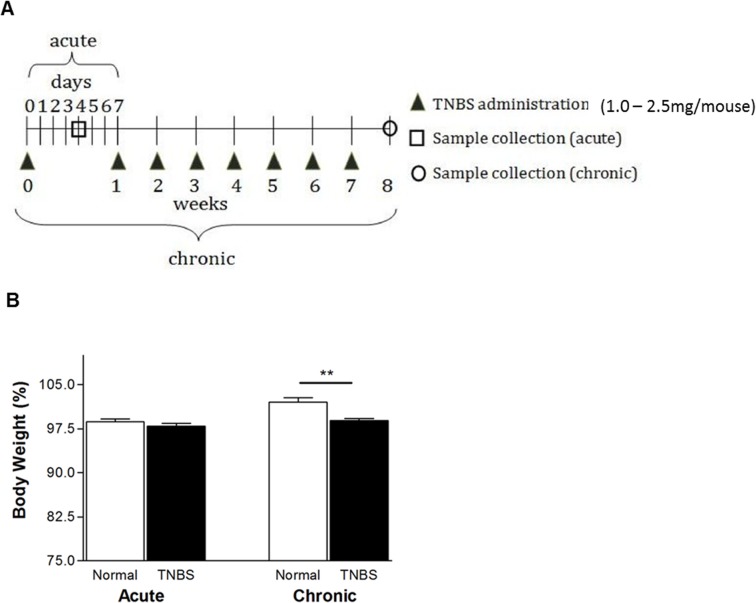
TNBS-induced acute and chronic colitis for Balb/c mice (n = 12). (A) Protocol. In acute colitis, mice received TNBS (1.0 mg in 45% ethanol) on day 0 and were sacrificed on day 4. In chronic colitis, mice received TNBS (1.0–2.3 mg) at one week intervals and were sacrificed on week 8. (B) Body weight changes measured through to week 8. Comparison of body weight between normal and TNBS on day 49, to determine ability to recover body weight, **p < 0.01

Experimental colitis was induced by intra-rectal administration of TNBS (Sigma-Aldrich, St. Louis. MO). In acute colitis, Balb/c mice received 1 mg of TNBS and C57BL/6J mice received 2.5 mg of TNBS at day 0 [(Balb/c: n = 12 (normal), n = 16 (TNBS); C57BL/6J: n = 9 (Cav-1^-/-^ TNBS), n = 12 (wild-type TNBS), n = 3 (Cav-1 -/- normal), n = 7 (wild-type normal)]. In Balb/c mice chronic colitis was induced by weekly administration of increasing doses of TNBS eight times (1.0–2.3 mg in 45% ethanol) as previously described [29, 30]. Mice were lightly anesthetized with isoflurane, and then intrarectally administered TNBS in 45% ethanol via a 3.5 F catheter affixed to a 1-mL syringe. The catheter was advanced into the rectum to a point 4 cm proximal to the anal verge, and TNBS was injected in a total volume of 100 μl. To ensure distribution of TNBS within the entire colon and cecum, mice were held in a vertical position for 50 seconds after the injection. Mice without TNBS administration served as controls. Four days (acute) and ten days (chronic) after the last TNBS administration, mice were sacrificed for sample collection (Fig. 1A).

### Body weight and clinical scores

In TNBS-induced colitis, starting body weight was measured prior to TNBS administration and weights were taken daily or weekly at the same time. Clinical scores were also determined at that time based on a combination of our experience and previously reported description [31]. Each mouse was observed and given a score (out of 20) based on appearance, behaviour, body condition, stool consistency, and rectal bleeding. Each item was graded from 0 to 4 as below: appearance (0: normal, 1: ruffled, 2: hunched, 3: closed eyes, 4: sunken eyes), behaviour (0: normal, 1: not moving, 2: depressed, 3: isolated, 4: hyperactive), body condition (0: emaciated, 1: under-conditioned, 2: well-conditioned, 3: over-conditioned, 4: obese), stool consistency (0: small, firm, dry, non-adherent and friable, 1: small, firm, moist, adherent, 2: large, soft, very adherent stool, 3: large, soft, pliable, 4: liquid stool)[31], and rectal bleeding (0: negative, 2: positive, 4: gross bleeding) [31].

### Histological examination

Colon sections were fixed in 10% buffered formalin and embedded in paraffin. Paraffin-embedded colon sections were cut (6-μm), stained with hematoxylin and eosin (H&E) and examined using an Olympus IX51 light microscope equipped with CCD camera under control of Image Pro Plus software (Media Cybernetics, Inc., Bethesda, MD). The sections of each group of mice were assigned a random code to blind the examiners. Scores determined by severity of inflammation 0–3; depth of injury 0–3; and crypt damage 0–4, as previously reported [32].

### Preparation of colon tissue extracts

Frozen colonic samples were mechanically homogenized in buffer containing 1M Tris-HCl, 3M NaCl, and 10% Triton supplemented with protease cocktail inhibitors (Sigma-Aldrich, St. Louis. MO). Samples were then frozen (-70°C) and thawed (37°C) three times, followed by centrifugation at 14,000 *rpm* for 30 min at 4°C. Supernatants were frozen at −70°C until assay.

### Measurement of cytokines by enzyme linked immunosorbent assays (ELISA)

Levels of TNF, IL-17, and IFN-γ in colon tissue extracts were measured by ELISA according to the manufacturer’s instructions (BD Bioscience, Franklin Lakes, NJ). The cytokine amount in the extract was normalized, representing the amount of 100 milligrams of tissue proteins measured by Bio-Rad protein assay.

### Measurement of Cav-1 levels by ELISA

To quantitatively measure Cav-1 levels, a sandwich ELISA was developed in which two commercial purified polyclonal anti-Cav-1 antibodies were chosen. The capture antibody was rabbit anti-human Cav-1, generated from human recombinant Cav-1 (#610060, BD Transduction Laboratories, Franklin Lakes, NJ). The detection antibody was an HRP-conjugated rabbit anti-human Cav-1 antibody, raised against a peptide mapping at the N-terminus of Cav-1 of human origin (#sc-894-HRP, Santa Cruz Biotechnology, Santa Cruz, California). To estimate relative amount of Cav-1 and to avoid variations among assays, an in-house Cav-1 standard extract was prepared by pooling various samples known to have high levels of Cav-1 and defined as 1000 units/ml. The standard extract was aliquoted and stored at −85°C. Negative controls were taken from Cav-1 knock-out mice, where no Cav-1 levels were detectable.

Costar microwell plates were coated with 1 μg/well of capture antibody in 50 mM carbonate/bicarbonate buffer, pH 9.6 (50 μl/well) and incubated overnight at 4°C. After three washes with 0.02 M phosphate buffered saline (PBS) containing 0.05% Tween 20 (pH 7.4), the wells were blocked with PBS containing 2.0% w/v BSA for 90 minutes at room temperature. Plates were washed once and samples (50 μl/well; 1:100 dilution with 0.2% BSA PBS) and standard extract dilutions (starting 1:20, 2-fold diluted for 11 dilutions) were added and incubated overnight at 4°C. Plates were washed three times and 50 μl of detection antibody, diluted 1:2000 in 0.2% BSA PBS, were added. After incubation for 90 minutes at 37°C, plates were washed five times and 50 μl/well of 3,3’,5,5’ Tetramethylbenidine (TMB) liquid substrate system for ELISA (Sigma-Aldrich, St. Louis. MO; T 0440) was added. Plates were incubated at room temperature, in the dark, for 20 minutes. The absorbance was immediately read at 370 nm. The value of Cav-1 was calculated by interpolation from the dilution curve of the standard extract and normalized, representing the amount of 100 milligrams of tissue proteins.

### Soluble collagen assay

Colon sections were homogenized in 0.5 M acetic acid containing 1 mg of pepsin (at a concentration of 10 mg of tissue/5 ml of acetic acid solution). The resulting mixture was then incubated and stirred for 24 h at 4°C. Total soluble collagen content of the mixture was then determined with a Sircol Collagen Assay Kit (Biocolor) [33]. Acid soluble type I collagen supplied with the kit was used to generate a standard curve. The results were normalized, representing the amount of 100 milligrams of tissue proteins.

### Statistical analyses

Values were expressed as mean ± SD. Differences between experimental groups were assessed by one-way analysis of variance (ANOVA) followed by Newman-Keuls multiple comparison test, or unpaired t-tests (GraphPad Prism). *P* values < 0.05 were considered statistically significant.

## Results

### In TNBS-induced colitis, body weight loss and colon inflammation are significantly increased (Balb/c mice)

In acute colitis, on day 4, significant body weight changes were not seen between normal and colitis mice, as obvious body weight loss usually occurs between days 1–3 after TNBS administration. But, in chronic colitis, as expected, mice receiving TNBS administration(s) showed significantly increased clinical scores (not shown) and body weight loss (102.3% vs. 99.8%, normal vs. TNBS in chronic colitis; *P* <0.01) (Fig. 1B).

**Fig 2:**
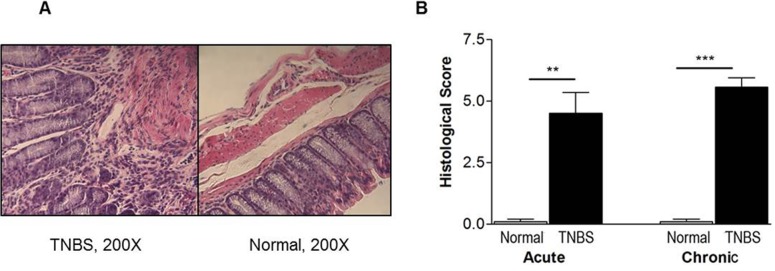
Histological analysis of colon sections stained with H&E in TNBS-induced colitis (n = 12). (A) Representative pictures in chronic colitis. (B) Semi-quantitative evaluation. Colon sections were semi-quantitatively evaluated double-blindly by a pathologist. Scores determined by severity of inflammation 0–3; depth of injury 0–3; and crypt damage 0–4. **p < 0.01; ***p < 0.001

In both acute and chronic colitis, mice receiving TNBS also had significantly increased colon inflammation. Fig. 2A shows represented colon inflammation images in chronic colitis, which include distorted tissue architecture, inflammatory cell infiltrates and goblet cell reduction. Semi-quantitative analysis showed the difference between colitis and normal mice was statistically significant (*P* < 0.01 in acute phase and *P* < 0.001 in chronic phase) (Fig. 2B).

### Cav-1 levels are reduced while levels of inflammatory cytokines and collagens are elevated in TNBS-induced chronic colitis (Balb/c mice)

**Fig 3:**
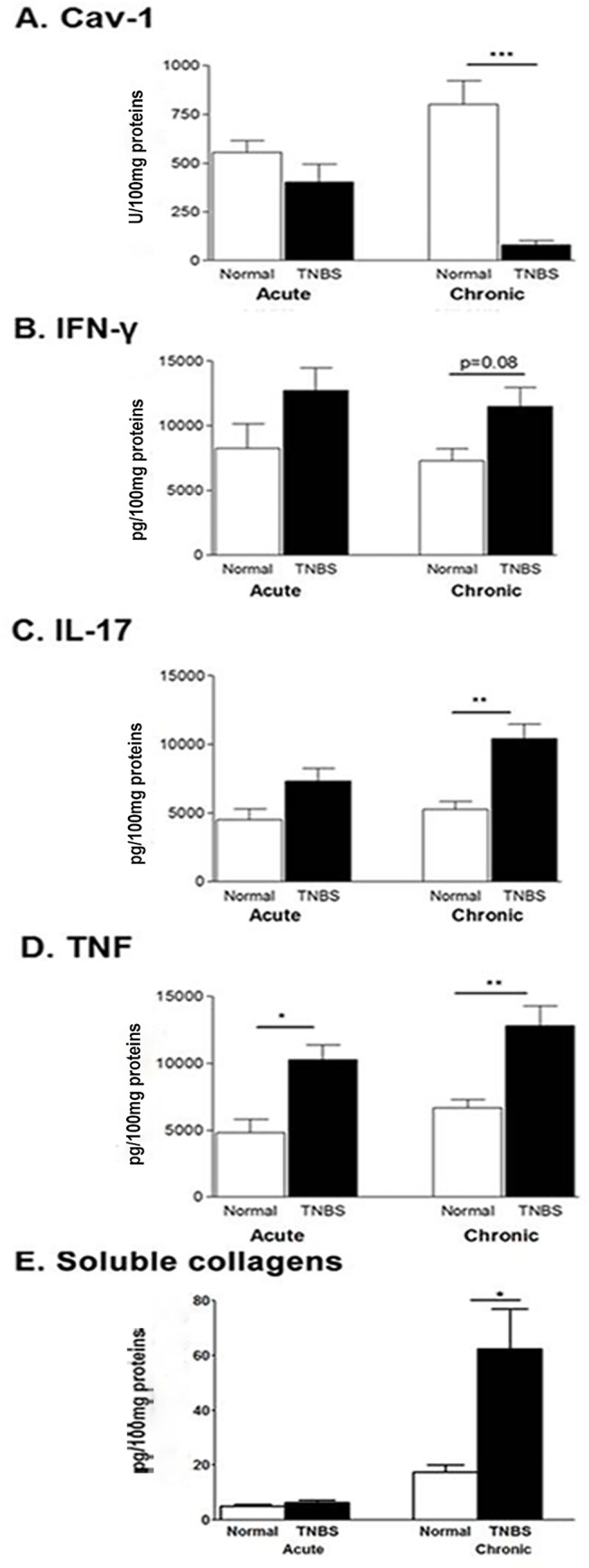
Colon caveolin-1, cytokine levels and soluble collagens in TNBS-induced colitis (n = 12). (A) Cav-1 levels detected by ELISA. (B) (C) (D)). Levels of IFN-γ, IL-17 and TNF measured by ELISA. (E) Soluble collagens measured by a Sircol Collagen Assay kit. Average of duplicate values taken. *p < 0.05 **p < 0.01; ***p < 0.001

To explore the relationship of Cav-1 and inflammatory cytokine levels, the levels of Cav-1 and inflammatory cytokines IFN-γ, TNF and IL-17 in colon tissue extracts were determined in mice with acute and chronic TNBS-induced colitis, using ELISA. To our surprise, mice receiving TNBS with chronic colitis had significantly lower levels of Cav-1 than normal mice (*P* < 0.0001) (Fig. 3A), although the changes in acute colitis were not significant. For all three cytokines assayed, mice that received TNBS showed increased levels when compared with normal mice (*P*’s < 0.01 for TNF and IL-17 levels and *P* < 0.05 for IFN- γ in chronic colitis) (Fig. 3B,C,D). In acute colitis, elevated cytokine levels were not significantly higher compared to normal mice. As fibrosis is a characteristic of chronic inflammation, soluble collagens in colon extracts were quantitatively measured. The results showed that the amounts of collagen were significantly increased in mice with chronic colitis, confirming the establishment of chronic colitis.

### Cav-1 levels inversely correlate with colon inflammation and cytokine levels

**Fig 4:**
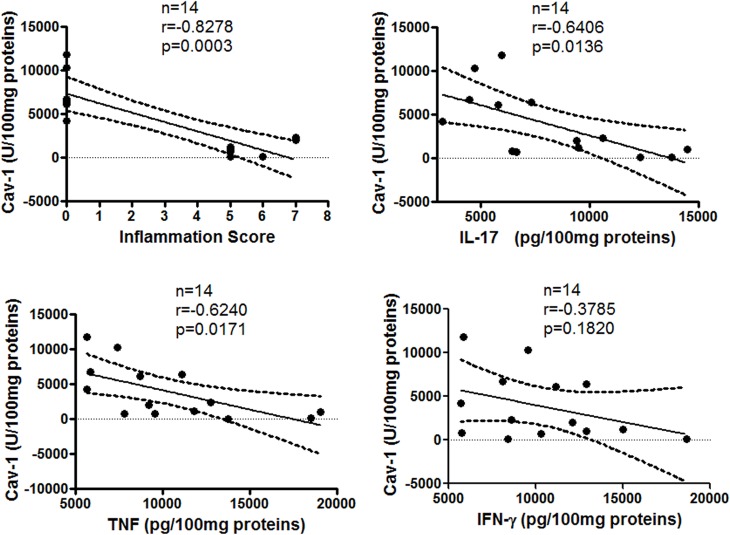
Correlations of colon Cav-1 levels with inflammation and cytokine levels in chronic colitis model.

To determine the correlation between colon Cav-1 levels and inflammation or cytokine levels in acute and chronic colitis, a correlation analysis was performed. In chronic colitis, colon Cav-1 levels were significantly and inversely correlated with colon inflammatory scores in (*P* < 0.0003, r = -0.828) (Fig. 4), and also inversely correlated with IL-17 and TNF, but not IFN, levels (*P* < 0.01; r = -0.641 for IL-17; *P* < 0.02, r = -0.624 for TNF). However, statistically significant correlations were not found in acute colitis (data not shown).

### Genetic deletion of Cav-1 results in increased clinical symptoms in TNBS-induced colitis (C57BL/6J mice)

**Fig 5:**
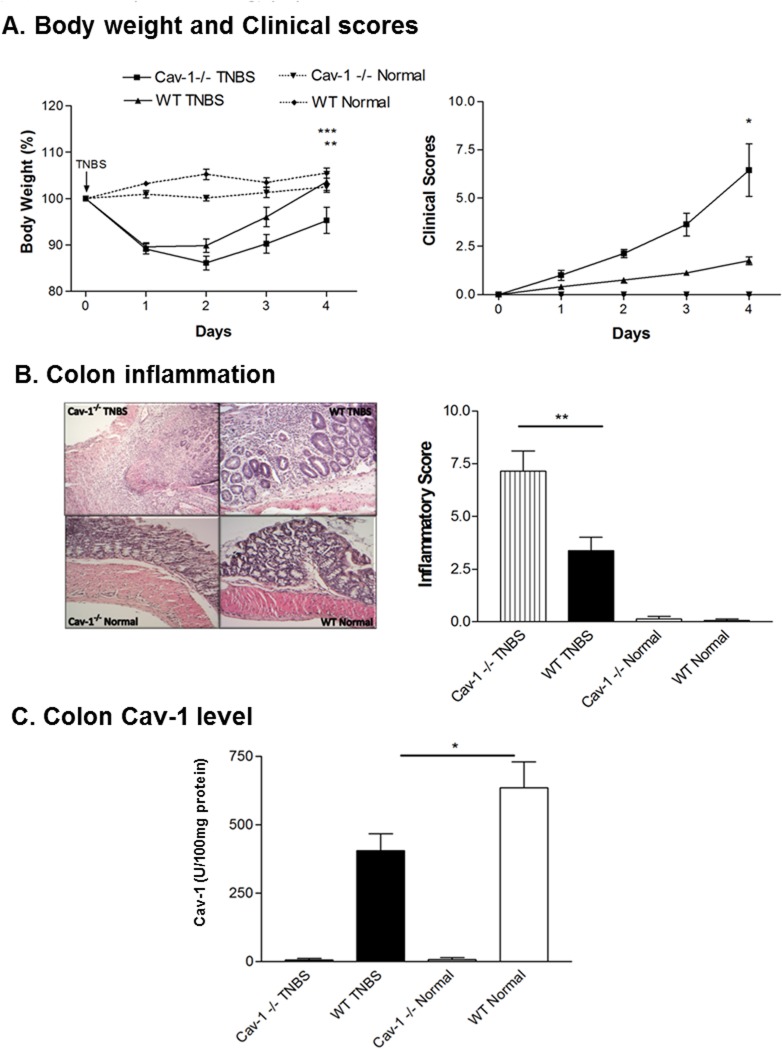
Acute TNBS-induced colitis for C57BL/6J Cav-1 knockout mice. (A) Body weight and clinical scores. (B) Colon inflammation. (C) Cav-1 levels determined through ELISA. Average of duplicate values taken. n = 9 in Cav-1-/- TNBS, n = 12 in wild-type TNBS, n = 3 in Cav-1-/- normal, n = 7 in wild-type normal. *p < 0.05; **p < 0.01; ***p < 0.001

To further confirm our findings that Cav-1 plays a protective role in TNBS-induced colitis, mice genetically deficient in Cav-1 (Cav-1^-/-^) underwent induction of acute TNBS-induced colitis. As shown in Fig. 5A, Cav-1^-/-^ mice receiving TNBS have increased body weight loss and higher clinical scoring, when compared with wild-type mice receiving TNBS (Fig. 5A). Also, histological analysis revealed that these mice had more severe colonic inflammation including inflammatory cell infiltration and distorted tissue architecture (which was confirmed by semi-quantitatively analysis (*P* < 0.01), compared with their wild-type counterparts (Cav-1^+/+^) receiving TNBS (Fig. 5B). As shown in Fig. 5C, ELISA results show that Cav-1^-/-^ mice have non-detectable Cav-1 levels.

Taking together, the above data indicate that Cav-1 may actually play a protective role in modulating disease activity and tissue inflammation in murine TNBS-induced colitis.

### Cav-1 levels are decreased in TNBS-induced colitis of C57BL/6J mice

To determine, whether Cav-1 levels were different in different species, wild-type C57BL/6J mice received one dose of TNBS administration. Colonic Cav-1 levels were assayed. The results were the same as those in TNBS-induced colitis in Balb/c mice. As shown in Fig. 5C, Cav-1 levels are significantly decreased in wild-type C57BL/6J mice receiving TNBS (WT TNBS) when compared to wild-type normal mice (WT Normal) (*P* < 0.05).

## Discussion

Employing a TNBS-induced murine colitis model, we induced both acute and chronic colitis in Balb/c mice and acute colitis in C57BL/6J Cav-1^-/-^ and wild-type mice. To quantitatively determine colonic Cav-1 levels, we developed a sandwich ELISA. We found that colonic Cav-1 levels were reduced in mice with TNBS-induced acute (statistical significance was not reached) and statistically significantly reduced in chronic colitis in both Balb/c (Fig. 3A) and C57BL/6J (Fig. 5C) mice. Mice receiving TNBS had significantly decreased Cav-1 levels (*P* < 0.0001 in chronic colitis) compared to that reported in the DSS-induced colitis model [26]. Further investigation showed that Cav-1 levels inversely correlated significantly with colonic inflammation and levels of inflammatory cytokines IL-17 and TNF (*P’s < 0*.*02)* (Fig. 4). Thus, Cav-1 might actually play a protective role in TNBS-induced colitis, which deserves to be explored further.

To confirm our findings in TNBS-induced colitis, Cav-1 knock-out mice were used to determine what effect the complete loss of caveolin-1 would have on inflammation in TNBS-induced experimental colitis. Mice lacking Cav-1 (Cav-1^-/-^) and receiving TNBS showed decreased body weight recovery and increased clinical scores, compared to wild-type mice receiving TNBS (Cav-1^+/+^) (Fig. 5A). After H&E staining, colon sections were semi-quantitatively evaluated blinded, by a pathologist. Cav-1^-/-^ mice receiving TNBS showed signs of more severe colon inflammation than control wild-type mice (Cav-1^+/+^) receiving TNBS (Fig. 5B). This supports our previous findings, suggesting that Cav-1 might play a protective role in TNBS-induced intestinal inflammation.

This protective role could be due to the involvement of Cav-1 in cellular signalling. During caveolae-dependent signalling, Cav-1 acts as a scaffold protein—it collects and organizes various signalling complexes involved in diverse cell activities [9]. As a result, removing or mutating caveolin-1 may affect a number of disease processes. Cav-1 has been shown to have several anti-inflammatory effects: through the regulation of endothelial nitric oxide synthase (eNOS), which is involved with the hyperaemia and permeability changes association with acute inflammation [34]; by inhibiting inflammatory mediators and promoting anti-inflammatory cytokines through alveolar macrophages [35]; or by suppressing airway smooth muscle cell proliferation and orchestrating receptor-mediated signal transduction that regulates phenotype expression of airway smooth muscle cells [36]. Studies have also reported that Cav-1 protects against sepsis by modulating the inflammatory response, alleviating bacterial burden, and suppressing thymocyte apoptosis [13].

Studies have also indicated that Cav-1 has an inhibitory effect on cytokine production through different mechanisms [15]. It has been widely accepted that Crohn's disease is caused by an overly aggressive Th1 and Th17 immune response [3]. The IL-23/Th17 pathway is also critical for the development of chronic intestinal inflammation [37]. Recent studies demonstrate Cav-1’s involvement with Toll-like receptor 4 (TLR4) in peritoneal macrophages, strongly suggesting that regulation of TLR4 function may occur within caveolae or lipid raft microdomains [38, 39]. In fact, Wang et al [38], found Cav-1 binding motifs within the amino acid sequence of murine TLR4. Mutating this biding site abolished the interaction and reversed the inhibitory effect of Cav-1 on cytokine regulation (TNF, IL-6), indicating that Cav-1 is able to inhibit TNF production through the activation of TLR4. Thus, as we have seen in TNBS-induced experimental colitis, a decrease in Cav-1 levels results in a higher TNF output, possibly due to a loss of TLR4 regulation. In addition, experiments done by Tang *et al*, showed that TLR4 activation is required for IL-17-induced tissue inflammation and wasting [40]. Once again, since we saw an inverse correlation between Cav-1 and IL-17 levels, we may speculate that less Cav-1 results in reversed inhibitory effects through TLR4, therefore leading to the increase of IL-17. Studies have also reported that Cav-1 can inhibit TGFβ signaling and reduced Cav-1 expression is associated with the activation of TGFβ signaling [16]. Taken together, the above studies of Cav-1’s anti-inflammatory effects support our findings in TNBS-induced colitis.

In a previous report, in DSS-induced mouse colitis, compared with normal mice, Cav-1 levels were increased, and genetic deletion (Cav-1^-/-^ mice) or pharmacologic inhibition of Cav-1 significantly decreased vascular density and angiogenesis scores [26]. Both this previous study and our current studies used Cav-1 knockout mice to explore the role of Cav-1 in colitis. The only difference between the two studies is the different type of mouse models of colitis used. There are possible explanations for these opposing findings of the role of Cav-1 in experimental mouse colitis in the two studies. First, the animal models used are different. IBD is a complex interaction of genes, environment, and intestinal flora. Much of what we know about IBD is gathered from our use of animal models that, although having similar characteristics of IBD, have their differences in pathogenesis. TNBS-induced colitis and dextran sodium sulphate (DSS)-induced colitis, are commonly used in studies, often without significant conflicting results [41–43]. However differences exist. Intrarectal delivery of TNBS induces colitis by haptenation of colonic proteins, leading to a delayed-type hypersensitivity reaction by causing Th1 and Th17 responses. This TNBS model is useful to study T cell-dependent mucosal immune responses, such as Crohn’s disease [44]. Also, the 45% ethanol contained in TNBS solution can elicit epithelial injury and it is therefore possible that healing or bacterial translocation responses may be altered by loss of Cav-1 expression. On the other hand, drinking water containing DSS is directly toxic to gut epithelial cells of the basal crypts and therefore may affect the integrity of the mucosal barrier, similar to changes in ulcerative colitis [45]. As T- and B-cell deficient C.B-17 SCID or Rag1^−/−^ mice also develop severe colitis, the adaptive immune system most likely does not play a major role (at least in the acute phase) in this DSS-colitis model [46]. Hence, the acute DSS colitis model is a particularly useful one to study the involvement of innate immune mechanisms in colitis. In addition, in the two mouse models, distinctive disease-specific cytokine profiles are identified. TNBS-induced colitis exhibits heightened Th1/Th17 responses (increased IL-12 and IL-17) as the disease becomes chronic. In contrast, DSS-induced colitis switches from Th1/Th17-mediated acute inflammation (increased TNF-alpha, IL6, IL-17, and KC) to a predominant Th2-mediated inflammatory response in the chronic state [47]. Taken together, although in T cell-independent colitis, such as DSS-induced colitis, Cav-1 exacerbates the disease, as previously reported [26], in T cell-dependent colitis induced by TNBS, mice that lack Cav-1 (Cav-1^-/-^) actually have less severe inflammation compared to controls.

Second, Cav-1 is a very complex membrane protein. Evidence indicates that caveolin-1 function is cell context dependent, resulting in different roles in diseases depending on stage or type, which may explain differences even between the two DSS studies. Studies have shown that Cav-1 can play opposite roles in the same disease. Cav-1 can act as both a tumor suppressor and as a tumor promoter [48]. In breast cancer cells, Cav-1 expression is significantly lowered when compared with normal breast tissue [49]. This decrease in Cav-1 expression (mRNA and protein level) was also seen in gastric, colon, and ovarian cancer cell lines [50–52]. On the other hand, caveolin-1 expression was elevated in carcinoma of the thyroid [53], associated with tumor dedifferentiation in bladder cancer [54], and enhanced the invasive capability of lung cancer cells lines [55].

Cav-1 may positively or negatively influence the development of atherosclerosis, depending on the cell type and the metabolic pathways regulated by this protein. Endothelial-specific overexpression of caveolin-1 accelerates atherosclerosis in apolipoprotein E-deficient mice [23], whereas, PPARgamma1-induced caveolin-1 attenuates atherosclerosis in apolipoprotein E-deficient mice [56]. In the cardiovascular system, one study shows that Cav-1 induces cardio-protection through epigenetic regulation [25], however, in biventricular damaged rodents, Cav-1 knock-out mice displayed decreased damage as well as decreased transcript levels of the proinflammatory marker plasminogen activator inhibitor-1 [57].

In lung injury, Cav-1 switches roles depending on the stage of the disease. In the initial state of acute lung injury, Cav-1 contributes to polymorphonuclear neutrophil-mediated inflammation, vascular injury and non-cardiogenic pulmonary edema. Yet in the late stage, Cav-1 may be beneficial as a potential antifibrotic protein [15, 17].

Many factors have been suggested as the cause of IBD, which appears to be an extremely complex disorder with a combination of genetic and environmental factors, enteric flora, and immunological abnormalities contributing [3, 58]. Given the role that Cav-1 plays in a number of cytokine signaling pathways and the fact that it can positively and negatively influence various diseases, it is not surprising that our finding of a protective role of Cav-1 in one model of IBD is different to that previously reported in an alternative model[26].

## Conclusions

We have found that in TNBS-induced colitis, colon Cav-1 levels are reduced and inversely correlated with colon inflammation and pro-inflammatory cytokine levels significantly. Moreover, mice that completely lack Cav-1 (Cav-1^-/-^ mice) have more severe clinical symptoms and colonic inflammation than control mice (Cav-1^+/+^) after TNBS challenge. The above results suggest that Cav-1 may play an important role in the protection of TNBS-induced colitis, and that enhancement of Cav-1 may be beneficial to IBD treatment. More importantly, as the results are opposite to those previously reported, it raises a critical issue regarding the evaluation of a molecule or treatment approach in animals: varying models might produce opposite results as different mechanisms are involved.

To explore the role of Cav-1 in inflammatory bowel disease, studies of patients with Crohn’s disease or ulcerative colitis should be carried out to define whether colonic levels of Cav-1 are positively or inversely correlated with the severity of the disease and to determine the mechanisms involved.
